# Effect of Acute Exercise on P300 Event-Related Potential and Its Correlation With Blood Lactate Levels

**DOI:** 10.7759/cureus.93063

**Published:** 2025-09-23

**Authors:** Kumar Abhishek, Tarun Kumar, Pooja Sakshi, Pooja Kumar

**Affiliations:** 1 Physiology, Indira Gandhi Institute of Medical Sciences, Patna, IND; 2 Microbiology, Employees' State Insurance Corporation Medical College and Hospital, Patna, IND

**Keywords:** acute exercise, bdnf, cognition, lactate, n2p3 amplitude, p300 erp

## Abstract

Introduction

Acute exercise can transiently enhance cognition. Lactate produced during aerobic exercise may cross the blood-brain barrier and influence neuroplastic pathways, potentially modulating P300 event-related potential (ERP) indices.

Materials and methods

In this pre-post interventional study conducted at Indira Gandhi Institute of Medical Sciences, in Patna, India (IEC approval number: 767/IEC/IGIMS/2022), 50 healthy male participants (18-40 years) underwent exhaustive aerobic exercise (Bruce protocol). Blood lactate was measured using the Sensa Core Lacto Spark point-of-care testing (POCT) (Sensa Core Medical Instrumentation Pvt. Ltd., Hyderabad, India). Auditory P300 ERPs were recorded at baseline, immediately post-exercise, 10 minutes post-exercise, and 20 minutes post-exercise, using a four‑channel MEP Neurosoft system (Neurosoft, Ivanovo, Russia) with 10-20 montage (Fpz ground, Cz active, M1/M2 reference) and 0.01-30 Hz bandpass. The oddball paradigm used 1000 Hz standard and 2000 Hz target tones (probabilities 0.80/0.20; sound pressure level (SPL) 60 dB; 10 ms rise/fall; 50 ms plateau). Outcomes included P300 latency, N2P3 amplitude, mean reaction time (MRT), true-click %, and false-click %. Analyses included repeated‑measures ANOVA with Bonferroni post hoc tests, Shapiro-Wilk for normality, Pearson correlation, and linear regression (IBM SPSS Statistics for Windows, Version 29.0.2.0 (IBM Corp., Armonk, New York, United States)).

Results

Lactate rose from 1.89±1.34 mmol/L (pre) to 6.91±2.24 mmol/L (immediate post) and declined at 10 minutes (4.80±1.72) and 20 minutes (3.43±1.31). P300 latency decreased significantly immediately after exercise when compared to the baseline (F=12.385; p<0.001); N2P3 amplitude increased (F=16.070; p<0.001); MRT decreased (F=8.007; p<0.001). Pairwise comparisons showed significantly shorter latency at all post-exercise time points vs. baseline; N2P3 amplitude was higher immediately post-exercise and lower at 20 minutes vs. baseline. True-click % increased immediately post (p=0.040), and false-click % decreased at 10 minutes (p=0.023). Lactate rise correlated positively with N2P3 amplitude (Pearson p<0.05), but the regression model did not reach significance (R²=0.072; p=0.059).

Conclusion

Acute exhaustive exercise significantly enhanced cognitive efficiency, reflected by reduced P300 latency, increased N2P3 amplitude, and faster reaction times. The association between lactate rise and ERP changes highlights its potential role as a biomarker and therapeutic target for exercise-induced cognitive benefits. These findings open avenues for clinical translation, where lactate-focused interventions and structured exercise regimens may be explored with cognitive performance and support rehabilitation strategies.

## Introduction

Physical activity enhances mental and physical health [1]. Various experiments have stated that physical activity causes structural and functional changes in the brain, leading to improvement in cognition and plasticity [2]. It was revealed after the astrocyte-neuron lactate shuttle that lactate generated during aerobic exercise was carried to the brain and had an impact on several brain functions [3]. These include a range of functions, ranging from neuroplasticity, dendritic spine formation, and reversing disease pathology to much more [4]. It is currently being considered that the generation of lactate and its transportation to the brain are related to the positive effects of exercise. Because of this, lactate has emerged as a crucial area of study to investigate the advantages and implications of this molecule. 

It is well recognized that regular exercise benefits cognition and brain health across various age groups, including older adults [5]. This understanding aligns with the view that "exercise is the real polypill" because of the peripheral factors it stimulates that are generated by the organ [6]. Generally, the impacts of routine physical activity on the body of an individual are thought to stem from the cumulative impact of numerous acute responses to individual exercise sessions. It is plausible to suggest that even though the benefits of acute exercise for brain function are temporary, they can contribute to long-term brain health if repeated consistently through prolonged physical training. But little is known about the precise processes by which long-term exercise improves brain function, especially how short-term exercise affects these long-term advantages. Among the immediate responses to exercise, there is increasing evidence that the myokine lactate crosses the blood-brain barrier, boosting the production of brain-derived neurotrophic factor (BDNF), thereby promoting neurogenesis, learning, and memory [7].

Following the stimulation of a peripheral nerve, recordings of waveforms from the nervous system are known as evoked potentials, and their names are derived from the pathways that are stimulated [8]. They are widely employed in research and clinical settings. They quantitatively depict signal transmission in particular central nervous system pathways and involve crossing at least one central synapse. They are of two types: sensory evoked potential and motor evoked potential. A number of repetitions are elicited, recorded, and then averaged to cancel the background activity [9]. Being a non-invasive technique, they are commonly used for monitoring brain activity and neuronal excitability.

P300 event-related potential (ERP) is a sensory evoked potential used to assess cognitive processing [10]. They provide a precise index of cortical activity. P300 latency denotes the time required for processing, while P300 amplitude represents the allocation of attentional resources throughout the stimulus engagement [11]. P300 has been used to evaluate adults' and children's cognitive functioning [12]. An ERP is a method used to evaluate brain function in response to sensory stimulation (e.g., touch, sound, sight, etc.). It offers a broad picture of how well the brain functions concerning attention, memory, and information processing abilities [13]. ERP, or time-locked electroencephalogram (EEG) activity, aids in recording brain activity linked to the cognitive and sensory functions [14]. The effects of immediate physical activity and blood lactate levels on brain activity in terms of attention, memory, and information processing skills have been examined in this study using auditory ERP.

Nowadays, lactate is being studied as a treatment for a number of central nervous system conditions and trauma victims [15,16]. From being thought of as a metabolic waste product to having the potential to serve as a therapy for a variety of illnesses and the reason for cognitive benefits of exercise, it is no surprise that it has now become an important and necessary research topic. In spite of so many studies, the effect of lactate on the central nervous system is still not clear and warrants further studies. Accordingly, the present study aimed to examine the effects of a single bout of exhaustive aerobic exercise on P300 ERP measures in healthy young men and to evaluate whether these electrophysiological changes were associated with concomitant alterations in blood lactate levels.

## Materials and methods

Study design and setting

This pre-post interventional research was carried out between January 2023 and June 2024 in the Department of Physiology at Indira Gandhi Institute of Medical Sciences (IGIMS), located in Patna, India. Fifty healthy male participants aged 18-40 years were recruited. All participants provided written informed consent.

Sample size calculation

The sample size for this investigation was calculated using a standard formula that accounts for data variability and the desired level of precision: $\text{n}=\frac{\text{Z}^{2}\times\text{s}^{2}}{\text{E}^{2}}$. In this equation, n denotes the estimated sample size, Z corresponds to the Z-score for a 95% confidence level (1.96), s represents the standard deviation, and E indicates the permissible margin of error for the variables under study. Although the minimum required sample size was determined to be 12, the final sample was increased to 50 participants to enhance statistical reliability and accommodate potential data loss.

Procedure and data collection

All participants were healthy adult males aged 18-25 years, recruited from the local community. A detailed clinical history and baseline assessment were undertaken to exclude potential confounders such as neurological, psychiatric, cardiovascular, or metabolic disorders, current use of medications influencing cognition or exercise tolerance, and history of substance abuse. This ensured a homogenous study population and minimized bias in ERP outcomes. All participants signed a written informed consent form and received complete information about the study protocol before taking part in the research.

All study procedures were conducted in accordance with the ethical principles outlined in the Declaration of Helsinki and were approved by the Institutional Ethics Committee of Indira Gandhi Institute of Medical Sciences (approval number: 767/IEC/IGIMS/2022). Blood pressure, temperature, heart rate, and BMI were measured at baseline. A clinical examination, history, and questionnaires were used to screen the participants first. The participants were screened for physical activity using the Physical Activity Readiness Questionnaire (PAR-Q) (see Appendices) [17].

Before the test began, the subjects were required to sit and relax for half an hour. The Sensa Core Lacto Spark point-of-care testing (POCT) equipment (Sensa Core Medical Instrumentation Pvt. Ltd., Hyderabad, India) was used to measure the blood lactate level. After that, the electrodes were positioned on the subject's scalp in accordance with the international 10-20 protocol, and the P300 ERP was recorded. The MEP Neurosoft four-channel machine (Neurosoft, Ivanovo, Russia), at the neurophysiology lab of the Physiology Department at Indira Gandhi Institute of Medical Sciences, was used to carry out the analysis. The subjects were then asked to perform an aerobic exercise using the Medicaid Cardivision Stress testing system (Medicaid Systems, Punjab, India) in the form of running as per the Bruce protocol until exhaustion. Following the exercise, the blood lactate levels were recorded, and P300 ERPs were once more assessed. The measurement and recording were repeated again at 10 minutes and finally at 20 minutes after the execution of the physical activity. Evoked potentials were recorded at four time points, namely, baseline (pre-exercise), immediately post-exercise, 10 minutes post-exercise, and 20 minutes post-exercise, while the ambient temperature was maintained at 25°C for the appropriate stimulation duration. Each P300 recording was generated using a total of 100 auditory stimuli. When the evoked potentials were being recorded, the individuals were told to stay still.

The P300 component was generated using an auditory oddball methodology, where subjects listened to two distinct tones, that is, approximately 1000 Hz for the standard and 2000 Hz for the target, via headphones. Each subject's auditory sensitivity was assessed for both frequencies before gathering the electrophysiological data. These tones were randomly played with a two-second gap between them, at a sound pressure level (SPL) of 60 dB, including a 10 ms rise/fall time and a 50 ms steady state. The target tone was presented less frequently than the standard, with respective probabilities of 0.20 and 0.80. The subjects were required to press the handheld remote button on hearing the target tone, and each session consisted of 100 trials, which were averaged to generate the P300 waveform. To reduce noise in the data, participants were asked to stay calm, mentally noting the target tones when they occurred [18]. The electrode placement adhered to the international 10-20 system [19]. We used surface electrodes, affixing them to the forehead at Fpz for the ground electrode, the vertex at Cz for the active electrode, and the mastoids for the reference electrodes M1 and M2 (corresponding to the left and right mastoid, respectively), using Ten20 conductive paste and micropore tape. We ensured that the inter-electrode impedance was kept at or below 5 kiloohms before beginning the tests. The filtering was set to a bandwidth of 0.01-30 Hz.

Data analysis

Data analysis was carried out using IBM SPSS Statistics for Windows, Version 29.0.2.0 (IBM Corp., Armonk, New York, United States). All collected data were initially compiled in Microsoft Excel (Microsoft Corporation, Redmond, Washington, United States) and presented as mean±standard deviation (SD). A p-value of less than 0.05 was considered statistically significant. Appropriate statistical methods, including repeated measures ANOVA, Shapiro-Wilk test, Pearson correlation, and linear regression, were employed to evaluate the continuous variables.

## Results

Age, height, weight, and BMI did not significantly differ among the subjects, according to the Shapiro-Wilk test, as evidenced by the p-values greater than 0.05 (Table 1). These results provide a clear baseline profile of the participants and justify the application of parametric statistical tests in subsequent analyses.

**Table 1:** Anthropometric parameters of the study population The table illustrates the demographic and anthropometric characteristics of the study participants, including age, height, weight, and BMI, represented as mean±SD. Each parameter showed a normal distribution based on the Shapiro-Wilk test.

Parameters	Mean±SD	P-value (Shapiro-Wilk)
Age (in years)	22.2±1.64	0.105
Height (in meters)	1.67±0.08	0.141
Weight (in kg)	63.1±7.31	0.705
BMI	22.6±1.80	0.726

**Figure 1:**
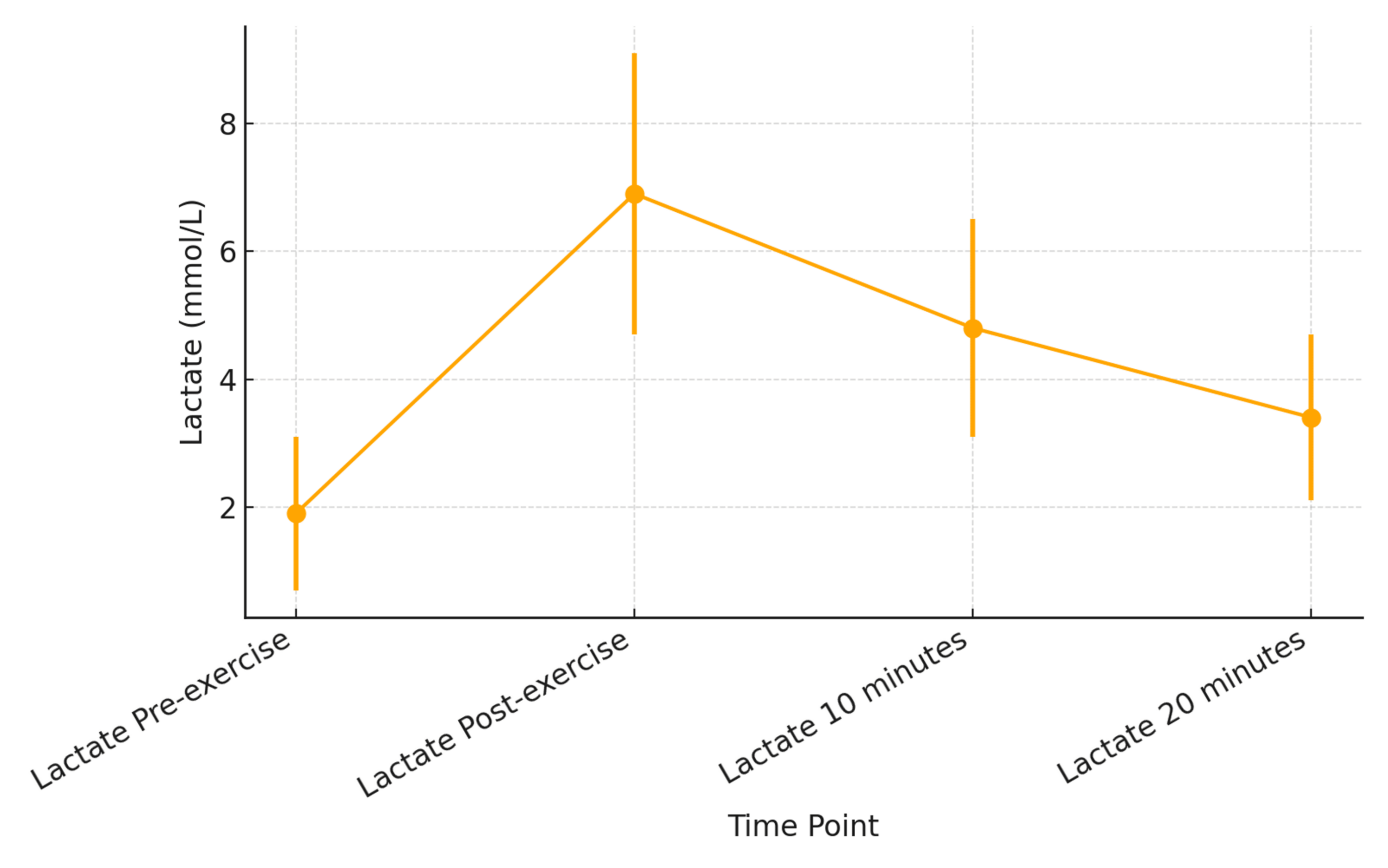
Blood lactate levels at different time intervals Line graph showing mean blood lactate concentrations (mmol/L) at four time points: pre-exercise (1.89±1.34), immediately post-exercise (6.91±2.24), 10 minutes post-exercise (4.80±1.72), and 20 minutes post-exercise (3.43±1.31). Data points represent the mean values for each time point (n=50), with error bars indicating ± standard deviation. Lactate levels show a marked rise immediately after exercise, followed by a gradual decline toward baseline over the subsequent 20 minutes.

Blood lactate levels rose significantly immediately after exercise and gradually declined over the next 20 minutes, although they remained elevated compared to baseline, as shown in Figure 1.

The P300 latency, N2P3 amplitude, MRT, true click %, and false click % (mean±SD) recorded in participants (N=50) before the exhaustive exercise (pre), right after its conclusion (end), as well as 10 and 20 minutes after its end are shown in Table 2. To minimize potential practice effects from repeated task exposure, participants underwent a familiarization session with the auditory oddball paradigm before data collection, ensuring stable baseline performance and reducing learning-related confounds in the P300 parameters.

**Table 2:** P300 parameter variation at different intervals All values are expressed as mean±SD. P300 Lat. (ms): P300 latency in milliseconds; N2P3 Amp. (µV): N2P3 amplitude in microvolts; MRT (ms): mean reaction time in milliseconds; True Click (%): number of correct responses; False Click (%): number of incorrect responses

P300 parameters	Baseline (pre)	Immediate (end)	10 minutes	20 minutes
P300 Lat. (ms)	333.78±27.02	305.66±26.21	318.46±32.55	322.54±30.60
N2P3 Amp. (µV)	8.32±3.08	9.91±2.91	8.68±3.84	7.29±2.78
MRT (ms)	306.66±32.201	290.84±31.13	298.98±39.61	289.90±38.04
True Click (%)	92.78±10.93	95.37±8.90	94.99±9.39	93.99±10.13
False Click (%)	3.30±2.61	2.42±2.06	2.28±2.13	2.50±3.17

To examine variations in P300 parameters and lactate concentrations across the four assessment intervals, namely, before exercise, immediately after, 10 minutes post-exercise, and 20 minutes post-exercise, a repeated measures ANOVA was conducted. This statistical approach was utilized to address within-subject variability and to assess time-dependent effects on the outcome measures (Table 3). Following it, post hoc comparisons using Bonferroni correction were performed to identify the specific time points demonstrating statistically significant differences (Table 3).

**Table 3:** Tests of within-subject effects for ERP by repeated measures ANOVA All values are based on repeated measures ANOVA. * indicates statistically significant values (p<0.05). P300 Lat. (ms): P300 latency in milliseconds; N2P3 Amp. (µV): N2P3 amplitude in microvolts; MRT (ms): mean reaction time in milliseconds; True Click (%): number of correct responses; False Click (%): number of incorrect responses; ERP: event-related potential

	Type 3 sum of squares	df	Mean square	F-value	P-value
P300 Lat. (ms)	20214.940	3	6738.313	12.385	<0.001*
N2P3 Amp. (µV)	174.985	3	58.328	16.070	<0.001*
MRT (ms)	9246.775	3	3082.258	8.007	<0.001*
True Click (%)	202.133	3	67.378	1.168	0.324
False Click (%)	31.615	3	10.538	2.300	0.080

All values for P300 parameters are expressed as mean±SD (as applicable). The parameters were recorded pre-exercise, immediately after exercise, and 10 and 20 minutes post-exercise. * indicates statistically significant values (p<0.05) derived by repeated measures ANOVA test.

Bonferroni post hoc corrections were applied. Statistically significant values (p<0.05) indicate meaningful changes in ERP parameters at specific post-exercise intervals.

The pairwise comparisons for P300 latency show significant differences between P3 (baseline) and P3 Post (immediate post-exercise) (p<0.001), P3 (baseline) and P3 10 (10 minutes post-exercise) (p=0.002), and P3 (baseline) and P3 20 (20 minutes post-exercise) (p=0.039) (Table 4).

**Table 4:** Pairwise comparisons for ERP P3 wave latency All values are expressed as mean difference±standard error. The test applied was the Bonferroni post hoc correction. * indicates statistically significant values (p<0.05). ERP: event-related potential; P3: baseline; P3 Post: immediate post-exercise; P3 10: 10 minutes post-exercise; P3 20: 20 minutes post-exercise

Group A	Group B	Mean difference	Std. error	P-value
P3	P3 Post	28.120	3.532	<0.001*
P3	P3 10	15.320	4.746	0.002*
P3	P3 20	11.240	5.303	0.039*

The pairwise comparisons for N2P3 amplitude show significant differences between N2P3 (baseline) and N2P3 Post (immediate post-exercise) (p<0.001) and N2P3 (baseline) and N2P3 20 (20 minutes post-exercise) (p=0.009) (Table 5).

**Table 5:** Pairwise comparisons for ERP N2P3 amplitude All values are expressed as mean difference±standard error. The test applied was the Bonferroni post hoc correction. * indicates statistically significant values (p<0.05). ERP: event-related potential; N2P3: baseline; N2P3 Post: immediate post-exercise; N2P3 10: 10 minutes post-exercise; N2P3 20: 20 minutes post-exercise

Group A	Group B	Mean difference	Std. error	P-value
N2P3	N2P3 Post	-1.591	0.197	<0.001*
N2P3	N2P3 10	-0.357	0.432	0.412
N2P3	N2P3 20	1.026	0.377	0.009*

The pairwise comparisons for MRT show significant differences between MRT (baseline) and MRT Post (immediate post-exercise) (p<0.001) and MRT (baseline) and MRT 20 (20 minutes post-exercise) (p<0.001) (Table 6).

**Table 6:** Pairwise comparisons for ERP MRT All values are expressed as mean difference±standard error. The test applied was the Bonferroni post hoc correction. * indicates statistically significant values (p<0.05). ERP: event-related potential; MRT: mean reaction time; MRT Post: immediate post-exercise; MRT 10: 10 minutes post-exercise; MRT 20: 20 minutes post-exercise

Group A	Group B	Mean difference	Std. error	P-value
MRT	MRT Post	15.820	2.607	<0.001*
MRT	MRT 10	7.680	4.331	0.082
MRT	MRT 20	16.760	4.383	<0.001*

The pairwise comparisons for true click % show significant differences between True Click (baseline) and True Post (immediate post-exercise) (p=0.040) (Table 7).

**Table 7:** Pairwise comparisons for ERP true click % All values are expressed as mean difference±standard error. The test applied was the Bonferroni post hoc correction. * indicates statistically significant values (p<0.05). ERP: event-related potential; True Click: baseline; True Post: immediate post-exercise; True 10: 10 minutes post-exercise; True 20: 20 minutes post-exercise

Group A	Group B	Mean difference	Std. error	P-value
True Click	True Post	-2.596	1.232	0.040*
True Click	True 10	-2.214	1.894	0.248
True Click	True 20	-1.214	1.850	0.515

The pairwise comparisons for false click % show a significant difference between False Click (baseline) and False 10 (10 minutes post-exercise) (p=0.023) (Table 8).

**Table 8:** Pairwise comparisons for ERP false click % All values are expressed as mean difference±standard error. The test applied was the Bonferroni post hoc correction. * indicates statistically significant values (p<0.05). ERP: event-related potential; False Click: baseline; False Post: immediate post-exercise; False 10: 10 minutes post-exercise; False 20: 20 minutes post-exercise

Group A	Group B	Mean difference	Std. error	P-value
False Click	False Post	0.880	0.449	0.056
False Click	False 10	1.020	0.435	0.023*
False Click	False 20	0.800	0.578	0.172

Correlation with the rise in blood lactate levels following exercise yielded the following results (Table 9).

**Table 9:** Correlation between lactate rise and ERP parameters Pearson correlation and linear regression analyses were performed to assess the association between lactate rise and ERP parameters. * indicates statistically significant values (p<0.05). ERP: event-related potential; MRT: mean reaction time

Variable 1	Variable 2	Bayes factor	95% CI	Pearson correlation (p-value)	Linear regression
ERP P300 latency	Lactate rise	6.410	(-0.374, 0.153)	0.120	N/A
ERP N2P3 amplitude	Lactate rise	1.533	(0.001, 0.502)	<0.05*	0.059
MRT	Lactate rise	8.457	(-0.310, 0.217)	0.053	N/A
True click %	Lactate rise	6.663	(-0.173, 0.359)	0.113	N/A
False click %	Lactate rise	9.030	(-0.259, 0.274)	0.259	N/A

Pearson correlation revealed a statistically significant association (p<0.05) between the rise in blood lactate levels and the increase in N2P3 amplitude, indicating that higher post-exercise lactate may be linked to enhanced cortical resource allocation (Table 9). Following this, linear regression was performed for N2P3 amplitude, which yielded a near-significant trend (p=0.059); the model did not meet conventional thresholds for statistical significance (Table 10). Other ERP variables, including P300 latency, MRT, and click responses, did not show significant correlations with lactate levels (Table 9).

**Table 10:** Correlation and linear regression analysis between lactate rise and N2P3 amplitude Pearson correlation and linear regression were used to assess the relationship between lactate rise and N2P3 amplitude. N2P3: event-related potential N2P3 amplitude; Coefficient B: unstandardized regression coefficient

Variable 1	Variable 2	Pearson correlation	R square	Coefficient B	F-statistic	Regression p-value
Lactate rise	N2P3 amplitude	0.269	0.072	0.168	3.746	0.059

Pearson correlation revealed a strong positive association between the rise in blood lactate levels post-exercise and the increase in N2P3 amplitude. Although the regression model explained 97.5% of the variance (R²=0.975), the relationship did not reach statistical significance (p=0.059) (Table 10).

**Figure 2:**
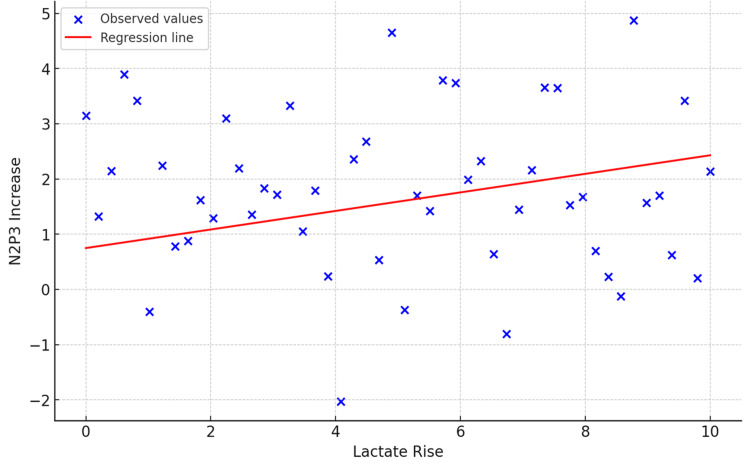
Scatter plot and linear regression between lactate rise and N2P3 amplitude increase The figure illustrates the relationship between lactate rise and N2P3 amplitude increase. Each blue cross represents an observed value, while the red line denotes the fitted linear regression model. A weak positive correlation was observed (r=0.269), though the association was not statistically significant (p=0.059).

Figure 2 illustrates the relationship between the increase in blood lactate levels following acute exercise and the corresponding increase in N2P3 amplitude. The red line shows the linear regression trend, and each blue cross represents a data point for a single patient. Although a positive association is visually evident, the relationship did not reach statistical significance (p=0.059) as shown in Table 10. This suggests that while lactate may influence cortical resource allocation (as reflected by N2P3 amplitude), the effect in this sample was modest and not strong enough to be conclusive. Further studies with larger sample sizes may help clarify this trend.

In our study, we found that after a brief short-term exercise, there was a significant rise in the blood lactate levels (Figure 1). We observed that the P300 latency decreased significantly immediately after the exercise (p<0.05), as evident in the ANOVA test (Tables 3-4). The ERP N2P3 amplitude increased significantly after the exercise. Pearson correlation showed that the rise in N2P3 amplitude was significantly correlated to the rise in the blood lactate levels, but the linear regression was not significantly correlated (Table 10). This means that changes in lactate rise do not significantly predict changes in N2P3 increase.

The MRT decreased immediately after the exercise, which was statistically significant (p<0.05); however, there was no significant correlation with the rise in blood lactate levels (Table 6). The true click % increased significantly immediately after the exercise, as evident in the pairwise comparison of the ANOVA test (Table 7). The false click % decreased significantly 10 minutes after the exercise, as evident in the pairwise comparison of the ANOVA test (Table 8).

## Discussion

Latency and amplitude are the primary metrics used to analyze the P300 wave, which has been suggested to represent the activity of the cerebral systems linked to the conscious perception of signals [20]. In our study, we found a highly significant (p<0.001) decrease in the P300 wave latency post-exercise. The latencies at 10 and 20 minutes were also statistically significant (p<0.05) when compared to the baseline. A decrease in the latency shows faster cognitive processing as observed in our study. In the case of N2P3 amplitude, also referred to as P300 amplitude, we found a highly significant increase immediately post-exercise (p<0.001). The P300 amplitude shows the allocation of attentional resources during stimulus processing; thus, an increase in the P300 amplitude shows a greater allocation of attentional resources, leading to an increase in the processing speed and cognitive power.

This is similar to the findings by Magnié et al., who in their study found significant P300 latency decrease and P300 amplitude increase in sedentary participants following a continuous progressive training program on a cycling ergometer [18]. Similarly, Kumar et al. in their study observed that acute moderate-intensity exercise caused a significant decrease in P300 latency and a significant increase in P300 amplitude among sedentary subjects [21]. Jain et al. conducted a similar experiment in which they measured the P300 latency of healthy adult male subjects before and after a continuous incremental exercise until volitional exhaustion [22]. They concluded that an intense exercise session improved the cognitive performance, as evident from a decrease in P300 latency and an increase in P300 amplitudes. This is similar to our observations. Yagi et al. in their study found that aerobic exercise led to a decline in the P300 latency but also led to a decreased P300 amplitude [23]. However, it is to be noted that their study design was different than ours. They recorded the P300 during the exercise, and both auditory and visual oddball tasks were recorded. This was opposite to the findings in other studies, and they reasoned that P300 amplitude in a primary task decreases when a secondary task requiring attention to another stimulus modality is added. This difference in the study design may have led to differences in the observations. Nakamura et al. in their study found a significant increase in the P300 amplitude post-exercise among well-trained joggers [24]. This is similar to the findings in our study and reflects that the beneficial effect of exercise on cognition is present in both sedentary and non-sedentary subjects. 

Pearson correlation between the rise in blood lactate level and the rise in P300 amplitude was found to be significant. Hence, based on this, we can assume that the increase in the resource allocation post-exercise, which is shown through the P300 amplitude, is associated with the rise in the blood lactate levels. However, the linear regression analysis showed a weak relationship between lactate rise and N2P3 increase, with the correlation not reaching statistical significance (p=0.059). This means that changes in lactate rise can affect N2P3 amplitude but do not significantly predict its increase. Gusatovic et al. conducted a detailed systematic review on the effect of acute exercise on ERP and cognition [25]. They concluded that an acute bout of moderate-intensity exercise increased P300 amplitude and decreased P300 latency, based on their evaluation of 52 peer-reviewed studies. They also commented that this increase in cognitive performance might be due to a post-exercise increase in BDNF levels. El Hayek et al. showed that lactate can activate the PGC1/FNDC5/BDNF pathway through SIRT1 and that voluntary exercise increased hippocampal BDNF expression and enhanced memory and learning in rats in a lactate-dependent manner [26]. SIRT1 activity in mice was stimulated by intraperitoneal lactate infusion, enhancing the PGC1/FNDC5/BDNF pathway and improving memory retention and spatial learning. Schiffer et al. explored whether lactate infusion at rest could raise BDNF blood concentrations in young adults using the lactate clamp method, which avoids stimulating physical exercise. They found that lactate levels and BDNF serum levels significantly increased after infusing a four-molar sodium-lactate solution and returned to baseline at follow-up, suggesting that the increase in BDNF serum post-lactate infusion might be driven by lactate expression or release from platelets due to blood gas disturbances [27]. Based on the above, we conclude that a rise in blood lactate level may be responsible for the improved cognitive efficiency post-exercise.

The reaction time associated with the P300 ERP component reflects the speed and efficiency with which the brain processes stimuli and responds to them. The MRT showed a highly significant decrease in duration immediately post-exercise (p<0.001). The true click % showed a significant increase immediately post-exercise (p<0.05), while the false click % showed a significant decrease at 10 minutes post-exercise. This shows an increase in accuracy post-exercise. A decrease in processing time and an increase in resource allocation might explain the faster reaction time and increase in accuracy post-exercise.

Itagi et al. in their study found a significant decrease in the reaction time among subjects post-exercise [11]. This is in line with our observations; however, they did find a decrease in the accuracy of responses as opposed to an increase in accuracy in our study. This may be due to the difference in the study design, as in their study, the subjects were to perform cognitive tasks like the Stroop test during ERP recording. Gusatovic et al., in their review, also observed that the reaction time post-exercise decreased and accuracy increased [25]. Similarly, in a study conducted by Won et al., they observed a faster reaction time among study participants after treadmill exercise [28]. However, the subjects for their study were trained soccer players. This suggests that the faster response and accuracy post-exercise are seen in both athletes and non-athletes. It further cements the notion that the beneficial effects of exercise are experienced by both sedentary and active personnel. 

Summing up, we observed that following acute exercise, there was a significant rise in blood lactate level, which was accompanied by a decrease in P300 latency, an increase in P300 N2P3 amplitude, a decrease in reaction time, and a significant increase in accuracy. Also, the increase in P300 amplitude showed a significant correlation to the rise in blood lactate level.

Limitations and scope for further research

The present study is not without limitations. First, the inclusion of only healthy adult male participants introduces a gender bias, thereby restricting the generalizability of the findings to the broader population, particularly females. Second, the study assessed the effects of lactate on cognitive ERP parameters only in the acute phase, without evaluating potential long-term or sustained changes, which may provide further insight into the enduring impact of lactate on cognition. Finally, while exercise-induced lactate rise was utilized in this investigation, future research should incorporate controlled lactate infusion studies to more precisely delineate its causal role in cognitive enhancement and to exclude confounding factors associated with exercise physiology. Addressing these limitations in subsequent studies may not only strengthen the mechanistic understanding of lactate's neuromodulatory effects but also facilitate its translation into therapeutic strategies aimed at optimizing cognitive performance.

## Conclusions

Based on the above finding, we conclude that acute exercise leads to significant enhancements in cognitive processing. Significant decline in P300 latency and the rise in N2P3 amplitude post-exercise suggest improved cognitive efficiency and a heightened allocation of attentional resources. The significant correlation between the rise in blood lactate levels and the increase in P300 amplitude further supports the hypothesis that lactate acts as a mediator in the neurobiological processes underlying cognitive improvements following exercise.

The benefits of acute physical activity on cognitive performance are further supported by the noted increases in accuracy and reaction time after exercise. While the exact mechanisms through which lactate influences cognitive processes remain to be fully elucidated, this study adds to the growing body of evidence suggesting that lactate is a key factor in exercise-induced cognitive enhancement. These results emphasize the potential of lactate as a therapeutic target for improving cognitive performance, which has significant implications for understanding how exercise promotes brain health and cognitive function. Further research is warranted to explore the long-term effects of repeated exercise bouts on cognition and to clarify the underlying mechanisms through which lactate exerts its effects on the brain. Also, future studies with larger, more diverse samples, the inclusion of female participants, and the direct measurement of BDNF would provide a more comprehensive understanding and further strengthen the conclusions.

## Figures and Tables

**Figure 3:**
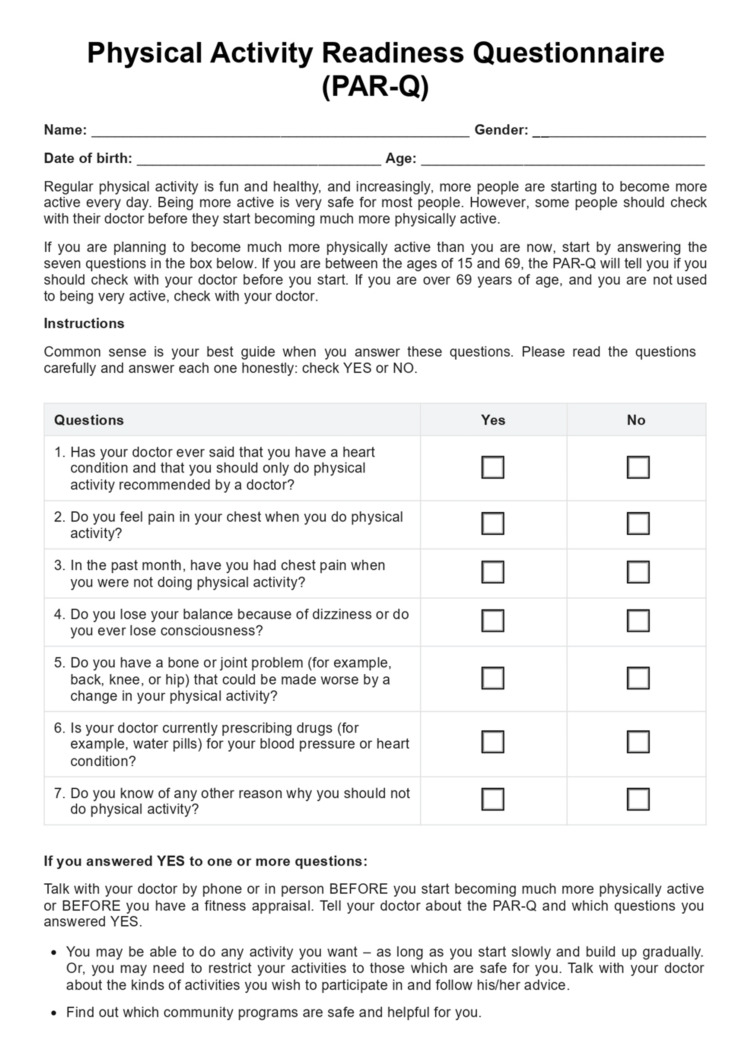
Physical Activity Readiness Questionnaire (PAR-Q)

**Figure 4:**
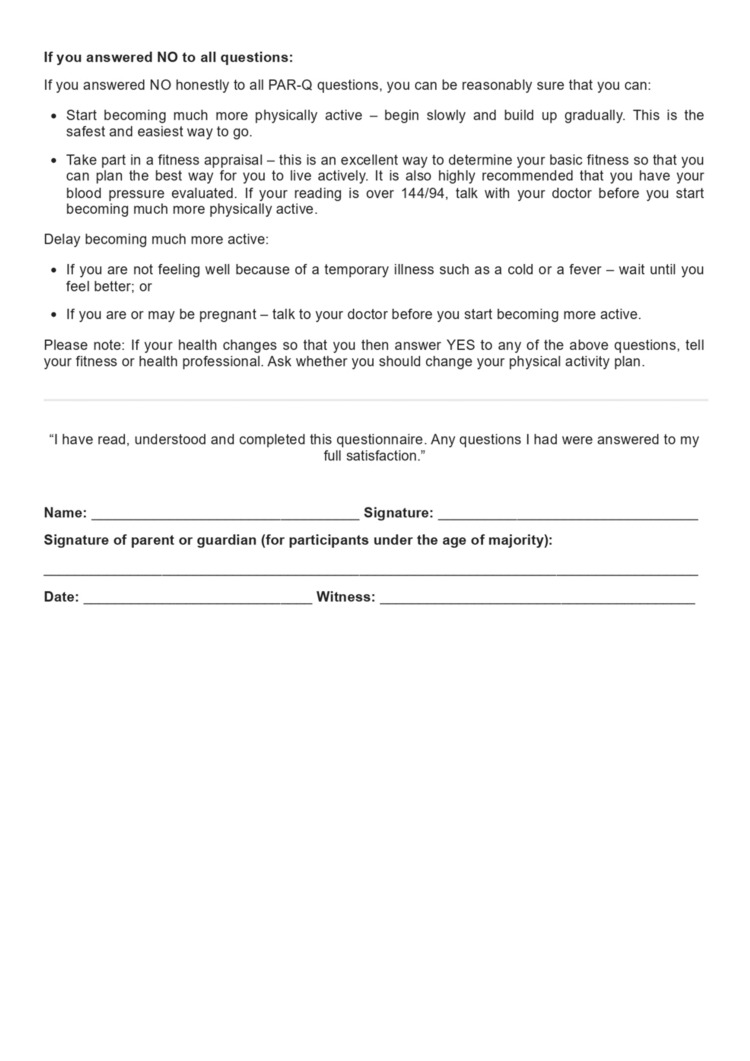
Physical Activity Readiness Questionnaire (PAR-Q) (continuation)
